# Harshening stem cell research and precision medicine: The states of human pluripotent cells stem cell repository diversity, and racial and sex differences in transcriptomes

**DOI:** 10.3389/fcell.2022.1071243

**Published:** 2023-01-04

**Authors:** Thong Ba Nguyen, Quan Lac, Lovina Abdi, Dipanjan Banerjee, Youping Deng, Yiqiang Zhang

**Affiliations:** ^1^ Department of Anatomy, Biochemistry and Physiology, Honolulu, HI, United States; ^2^ Center for Cardiovascular Research, Honolulu, HI, United States; ^3^ Department of Medicine, Honolulu, HI, United States; ^4^ Department of Quantitative Health Sciences, Honolulu, HI, United States; ^5^ Genomics and Bioinformatics Shared Resource, Honolulu, HI, United States; ^6^ Diabetes Research Center, John A. Burns School of Medicine, University of Hawaii at Manoa, Honolulu, HI, United States

**Keywords:** ethnicity, sex, human induced pluripotent stem cells (hiPSC), diversity & inclusion, transcriptomics (RNA-Seq), machine learning, network analysis

## Abstract

*In vitro* investigation on human development, disease modeling, and drug discovery has been empowered by human induced pluripotent stem cell (hiPSC) technologies that form the foundation of precision medicine. Race and sex genetic backgrounds have become a major focus of many diseases modeling and drug response evaluation in the pharmaceutical industry. Here, we gathered data from major stem cell repositories to analyze the diversity with respect to ethnicity, sex, and disease types; and we also analyzed public datasets to unravel transcriptomics differences between samples of different ethnicities and sexes. We found a lack of diversity despite the large sample size of human induced pluripotent stem cells. In the ethnic comparison, the White group made up the majority of the banked hiPSCs. Similarly, for the organ/disease type and sex comparisons, the neural and male hiPSCs accounted for the majority of currently available hiPSCs. Bulk RNA-seq and single-cell transcriptomic analysis coupled with Machine Learning and Network Analysis revealed panels of gene features differently expressed in healthy hiPSCs and human induced pluripotent stem cell-derived cardiomyocytes (hiPSC-CMs) of different races and sexes. The data highlights the current ethnic and sex inequality in stem cell research and demonstrates the molecular biological diversity of hiPSCs and cardiomyocytes from different races and genders. We postulate that future efforts in stem cell biology, regenerative and precision medicine should be guided towards an inclusive, diverse repository reflecting the prevalence of diseases across racial and ethnic groups and the sexes, important for both common and rare disease modeling, drug screening, and cell therapeutics.

## Introduction

The advent and rapid development of induced pluripotent stem cell (iPSC) fields have reshaped biological research and greatly enhanced toolkits for disease modeling, regenerative and precision medicine, biomedical engineering, and drug discovery. The conversion of somatic cells into iPSC state is accompanied by epigenetic remodeling, including DNA and chromatin modifications (Milagre et al., 2017), and reprogramming of microRNAs (Polo et al., 2012) and long non-coding RNAs (Kim et al., 2015), after which iPSCs shall closely resemble embryonic stem cells (ESCs). With fewer ethical concerns about ESC derivation and application, human iPSCs (hiPSCs) are now preferred models in numerous studies on basic biological functions such as differentiation and development, as well as disease modeling, precision/personalized regenerative medicine, and drug discovery. Nonetheless, genetic diversity of races and sexes poses natural challenges to hiPSC development and therapeutic implication (Lu and Zhao, 2013; Milagre et al., 2017; Doss and Sachinidis, 2019). Moreover, decreased diversity in samples suggests that the diversity of patients receiving clinical care is lost during the recruitment of clinic subjects into research (Kingswood et al., 2017).

There is an urgent need to understand the genetic basis for ethnic differences in cardiac, metabolism, and other functions and how it affects disease susceptibility among different ethnic groups in order to inform population-specific recommendations and personalized interventions for related disorders. Genetic disorders such as cardiovascular, diabetes, and neural diseases should have a diverse patient constituency. In this study, we analyzed the states of diversity in hiPSC repositories and dissected transcriptomics profiles in hiPSC and hiPSC-derived cardiomyocytes in different races and sexes. This study provides important information for the future development of diverse, inclusive iPSC lines and repository systems for disease modeling, drug screening, cell therapeutics, and precision medicine.

## Methods

### Study design

In this study, we gathered the data on sex and racial distribution of all primary hiPSC samples across normal (healthy) conditions and twelve main disorders from seven repositories: Boston University iPSC Bank, Cedar Sinai Medical, Corriel Institute, FujiFilm Cellular Dynamics-California Institute for Regenerative Medicine (CIRM), European Bank for Induced pluripotent Stem Cells (EBiSC), Mount Sinai Medical Center Stem Cell Repository, New York Stem Cell Foundation (NYSCF) Repository. Repository data from the providers were curated to remove duplicated cell lines based on the overlapping reference across the repositories, then unified for the essential entries such as sex, race/ethnicity, and disease type in the merged dataset. hiPSCs without race and sex information were assigned to “Other” and “No Report,” respectively.

The population difference among racial/ethnic groups is of our primary interest. Racial/ethnic designation includes the following groups: White, Black/African American, American Indian/Alaska Native, Asian, Hispanic or Latino, Native Hawaiian or Other Pacific Islander (NHPI), Mixed, and Other. In the adjusted analyses of examining population differences, the following covariates were included: sex and comorbid conditions (cardiovascular, pancreas, lung, liver, neural disease, cancer, or others). This study adheres to the guidelines of the University of Hawaiʻi at Mānoa Institutional Review Board. Data were collected until 1 May 2022.

### Study population

This study included 5,120 samples from seven databases of hiPSC.

For bulk RNA-seq and single-cell RNA-seq analysis, we used sample information and data from Mount Sinai Medical Center’s hiPSC repository, which is relatively balanced in the race and gender compositions (Supplementary Table S1) (Schaniel et al., 2021).

### Transcriptomic signature analysis

We utilized the bulk and single-cell RNA-seq (scRNA-seq) datasets of healthy hiPSCs and hiPSC-CMs from the previous study covering 40 hiPSCs with relatively balanced races and ethnicity (Supplementary Figure S1) (Schaniel et al., 2021). The related Gene Expression Omnibus (GEO) accession numbers are: GSE156384—bulk RNA-seq of duplicated samples of the 40 hiPSCs; GSE174773—bulk RNA-seq of hiPSC-CMs including 3 male (2 White and 1 Hispanic) and 3 female lines (2 White and 1 Asian); and GSE175761—scRNA-seq of CMs derived from 2 female and 2 male in White hiPSC lines. Supplementary Table S1 shows the sample information for these bulk- or single-cell RNA-seq data, and Supplementary Figure S1B depicts the general workflow of bioinformatics analysis.

Differential gene expression (DEG) of selected genes was assessed using the *Limma* package (Ritchie et al., 2015), with the *p*-values corrected using the FDR correction toolkit. Gene Ontology (GO) and Kyoto Encyclopedia of Genes and Genomes (KEGG) pathway through *Enrichr* tool (Xie et al., 2021) or the *clusterProfiler* package (Wu et al., 2021b). The t-test was used for the comparison of continuous characteristics between two groups, whereas the analysis of variance or chi-square tests was used for multiple groups. To test the differences between levels of skewed continuous variables, a non-parametric test of the trend was used. Statistics were prepared using R language (ver. 4.1.1; R Development Core Team; Vienna, Austria). All *p* values presented are two-sided; and significance testing used an alpha error level of less than 0.05.

### Machine learning

The maximum relevance minimum redundancy (mRMR) algorithm (Radovic et al., 2017; Bose et al., 2021), iteratively selects genes that are maximally relevant and minimally redundant for class prediction. The redundancy (correlation between genes) was calculated through the Pearson correlation coefficient, and the relevance (correlation between the class) was calculated by the F-statistic (regression). The gene importance score among the DEGs was calculated based on integrated relevance and redundancy information of each gene through *mrmr_classif* or *sklearn* toolkit in python. The score of each gene was ranked and visualized by *ggplot 2*.

Three classification algorithms, including support vector machine (SVM) (Guyon et al., 2002), logistic regression (Bowden et al., 2021), and naïve Bayes algorithms (Jiang et al., 2018) were used to investigate the validity of the ten mRMR genes. These algorithms have strong power, with supervised learning that carries out a binary classification of data (Maktabi et al., 2020). The advantages of these algorithms in predicting are quantitative and qualitative (Meeh et al., 2009). The accuracy and area under the ROC curve (AUC) were computed based on the ten hub genes of the hiPSC-CM transcriptomic dataset, respectively. The training set obtained 80 percent and the test set obtained 20 percent of the total samples for three classifier algorithms. In this study, mRMR and three classifier algorithms were conducted using Scikit-learn (*sklearn*) toolkit in Python 3.

### Regulatory network of TF-miRNA-mRNA in hiPSC-CM transcriptomes

This study conducted a network-based approach to explore the DEGs-TFs -miRNA interaction to detect the potential molecular regulatory signatures of the top ten hub DEGs after mRMR selection. To explore TFs that bind to regulatory regions of DEGs, significant TFs were attained from the JASPAR database (Khan et al., 2018). To investigate miRNAs that bind to target DEGs (mRNA) to negatively regulate their protein expression, significant miRNAs were deployed from TarBase (Karagkouni et al., 2018) and mirTarbase databases (Huang et al., 2020), and visualized through NetworkAnalyst (Xia et al., 2015), a topological analysis.

### Analysis of single-cell transcriptomics profiles in hiPSC-CMs

Single-cell RNA-seq dataset of healthy hiPSC-CMs was obtained from the Gene Expression Omnibus, accession No. GSE175761 (Schaniel et al., 2021). To integrate the four hiPSC-CM samples (2 females and 2 males White), we used the anchoring integration method implemented in R package Seurat v4.0 (Butler et al., 2018; Stuart et al., 2019), which is based on canonical correlation (CC) analysis.

The data was normalized using *LogNormalize* (natural log, by default setting), a global-scaling normalization method. The top 2000 variable genes were selected in each matrix and were used as input for the *FindIntegrationAnchors* function of Seurat (Hao et al., 2021).

The expression matrices were then integrated with the *IntegrateData* function. The integrated data were conducted principal component analysis (PCA; top 30 dimensions) to reduce dimension. In the PCA space, nearest neighbors were defined among cells with KNN method (*FindNeighbors*, top 30 PCs were selected), and cells were then grouped with Smart Local Moving (SLM) algorithm (*FindClusters* in Seurat, resolution equal to 0.5). Uniform manifold approximation and projection (UMAP) through *RunUMAP* function was used to visualize clusters with representative markers (Becht et al., 2018). Gene expression was visualized through the Seurat functions (*Vlnplot*, *DotPlot*, *heatmap* and *FeaturePlot*), respectively.

### Differential gene expression (DEG) analysis

*FindMarker* function in Seurat with the default Wilcoxon’s rank-sum test was used for DEGs between sex analysis (Hao et al., 2021). DEGs for scRNA-seq data were selected based on cutoff avg_logFC of 0.25, and at least 25% of cells expressed the markers.

### Weighted gene co-expression network analysis (WCGNA)

The genes depending on sex were explored by WCGNA analysis that was adapted from our previous study (Nguyen et al., 2021). Briefly, the value of the gene was used as an input for the analysis implemented in the WGCNA package (Zhang and Horvath, 2005). Detection of hub genes was based on the highest value of the gene significance (GS) (Wang et al., 2020), module membership (MM), and intramodular connectivity (K.in). The overlap of WCGNA hub genes with GS > 0.2 and MM > 0.5 in the best module and DEGs were selected as potential hub genes (Pol et al., 2017; Wu et al., 2021a). In summary, this method helped improve prediction and reduce dimensional issues.

### Gene-drug interaction analysis

The drug-gene interaction database (DGIdb) was used to discover potential drugs that interact with the top ten hub genes between sex single cell analysis. CYTOSCAPE software (Ver. 3.9.1) was used for drug-gene network visualization.

## Results

### Different races/ethnicities present with organ diseases

**FIGURE 1:**
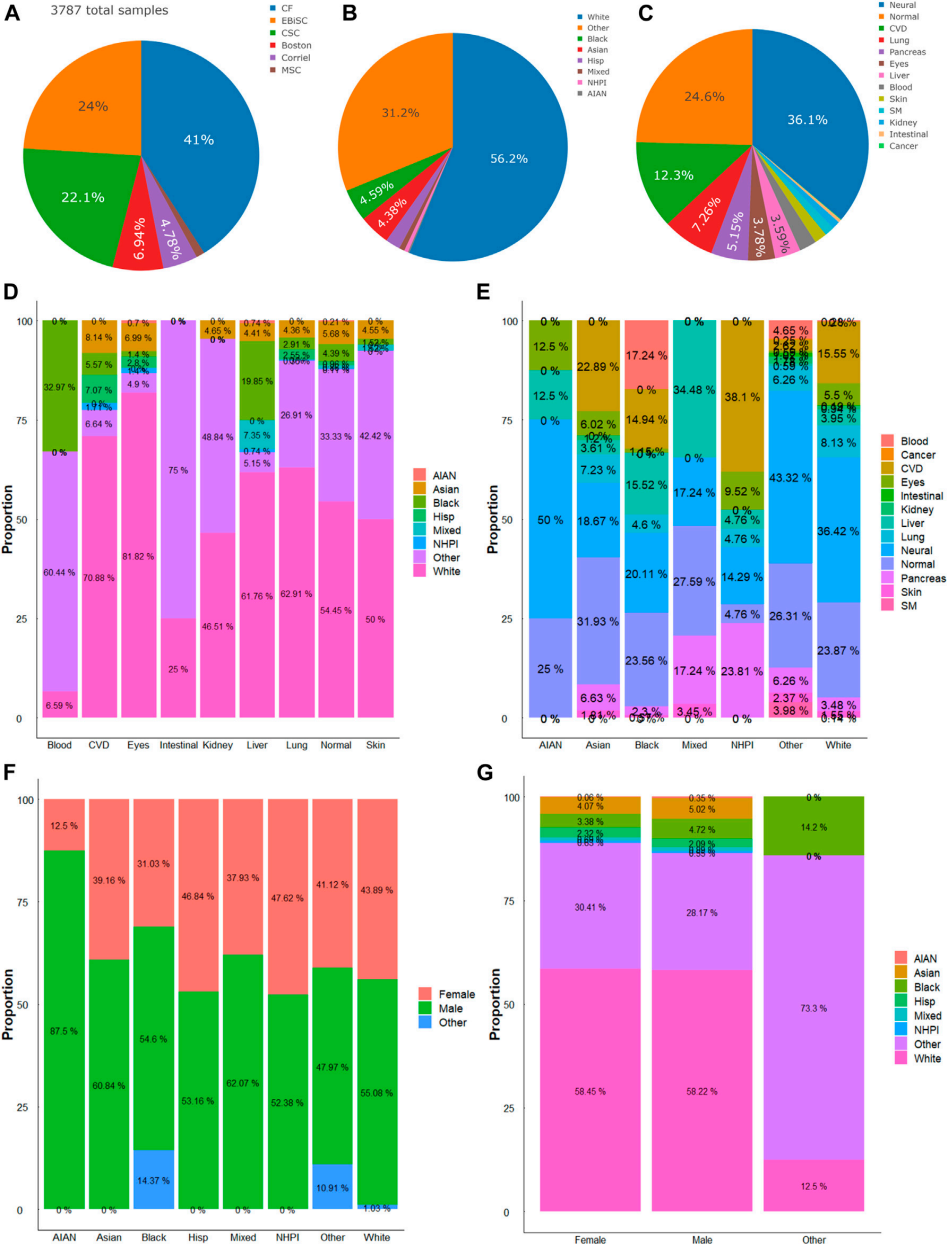
Compositions of hiPSC lines. **(A)** Six major repositories account for a total of 3,787 hiPSC lines included in this study. **(B)** Distribution of races and ethnicity of hiPSC lines. **(C)** Distribution of diseases or organ sources of hiPSC lines. **(D)** Proportions of races/ethnicities in hiPSCs of different diseases or organ sources. **(E)** Proportions of different diseases or organ sources in each race/ethnicity. **(F)** Proportions of sexes in hiPSCs of different races/ethnicities. **(G)** Proportions of race/ethnicity in hiPSCs of the female, male, or other (or unidentified) sex. AIAN, American Indian or Alaska Native; NHPI, Native Hawaiian or Other Pacific Islander.

We analyzed the state of the diversity of iPSC repository regarding sex, ethnic group, and targeted disease types. In the seven hiPSC banking repositories available in the United States (US) and Europe, a total of 5,120 lines were accounted for at the time of writing this report. We analyze NYSCF’s 1,333 line separately given the limited information from the provider (Supplementary Material). There were 3,787 hiPSC cell lines banked by the other 6 repositories, on which we performed more detailed analysis (Figure 1A). Among the available hiPSCs, more than a half were from male donors (male: 53.16% (*n* = 2013 hiPSC lines); female: 42.2% (*n* = 1,598); and others (not reported or abnormal): 4.6% (*n* = 176). In terms of race/ethnicity, the White cohort made up 56.2%, the other cohort accounted for about one-tenth of the total, and the unidentified (labeled “Other”) donors accounted for 31.2% (Figure 1B). About one-fourth of the hiPSCs were derived from healthy (normal) donors. And for hiPSCs in disease/organ types, neuronal disease was the major group (36.1%), followed by cardiovascular (12.3%), lung (7.26%), pancreatic (5.15%), eye (3.78%), and liver-related (3.59%); and those derived from blood, skin, skeletal muscle, kidney, intestine, and cancer, accounted for a minor faction so far (Figure 1C). And within disease/organ types of each race/ethnicity or *vice versa*, there were different levels of diversity (Figures 1D, E). It is worth noting that among the 21 Native Hawaiian or Other Pacific Islander (NHPI) hiPSCs, the majority were subjects with cardiovascular disease and diabetes.

### Different sexes present with unique races/ethnicities

There were more male hiPSC lines than female hiPSCs among the available repositories. The comparison within the races shows the most significant difference between the American Indian or Alaska Native (AIAN) group and the Black (African) cohorts. In the AIAN cohort, females comprised 12.5% of their samples, while males comprised 87.5%. And in the Black cohort, females comprised 31.03%, males comprised 54.6% and the remaining 14.37% are non-reported (Figure 1F). We then compared the different races within each sex cohort. The data showed that in both male and female groups, the White and the “Other” races comprised the majority of the samples (Figure 1G). In the female group, the White group makes up 58.45%, the other makes up 30.41%, and the other race groups make up the rest. There is a similar trend in male hiPSCs samples.

### Transcriptomics profiles in hiPSCs of different races and sexes

**FIGURE 2:**
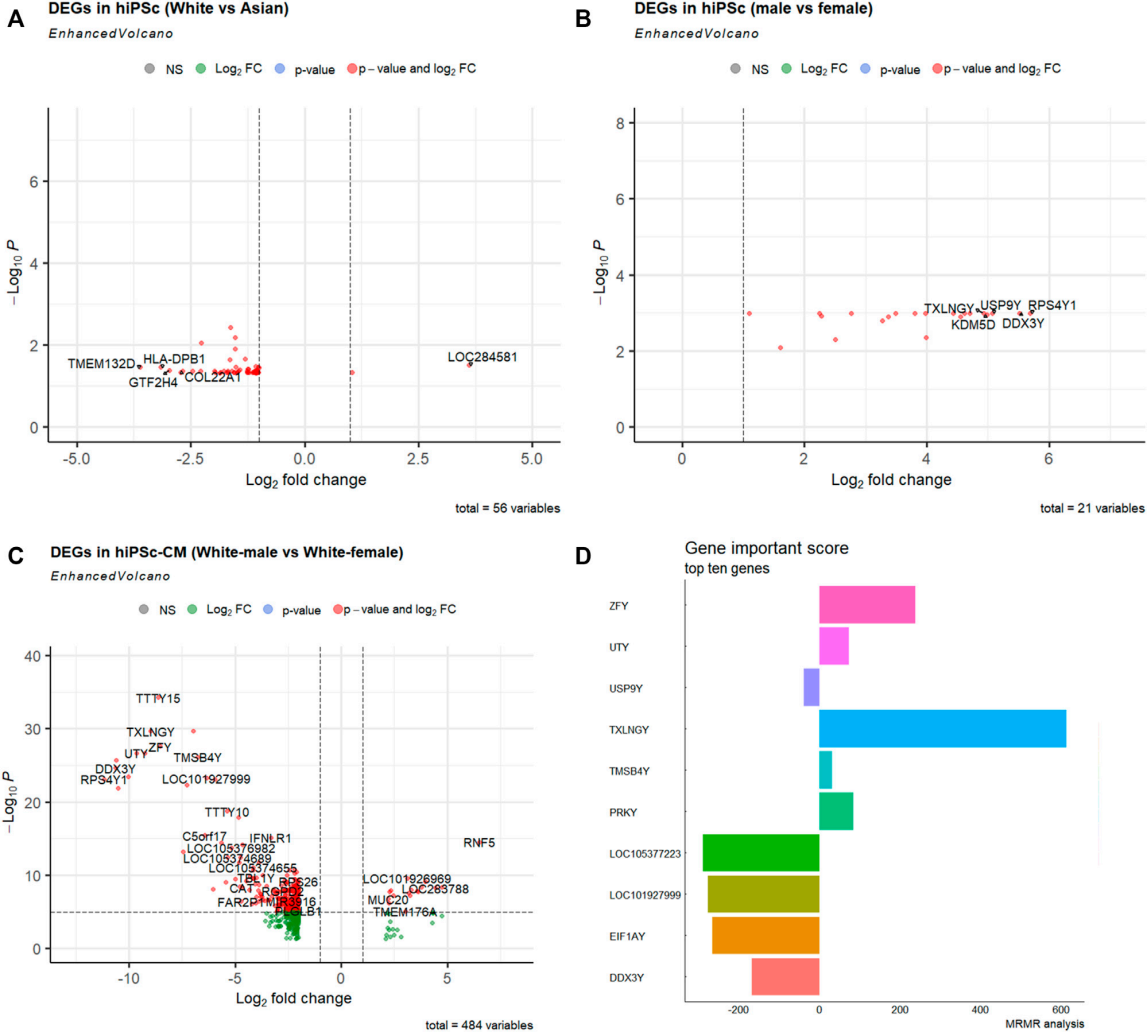
Differentially expressed genes (DEGs) in hiPSCs and hiPSC-CMs of different races and sexes. **(A)** Volcano map DEGs between White (*n* = 20) and Asian (*n* = 10) using combined male and female hiPSCs. **(B)** Volcano map DEGs between males (*n* = 19) and females (*n* = 18) using White, Asian, and Hispanic hiPSC datasets. **(C)** Volcano map of DEGs between White male and females hiPSC-CM (*n* = 2 for each sex). The *x*-axis represents the corrected *p*-value (scale conversion using logarithm), and the *y*-axis represents the fold change (log2FC). Each dot in the figure represents a gene; red or green dots represent genes that are significantly upregulated or downregulated, respectively. **(D)** Top ten gene importance scores between White male and White female hiPSC-CMs.

To compare the molecular signatures of hiPSCs among races, we utilized the RNA-sequencing data from Mount Sinai Medical Center’s recent stem cell repository that was relatively balanced, and composed 4 ethnic groups: Asian (6 females; 4 males), White (11 females; 9 males), and White-Hispanic (3 females; 6 males) (Supplementary Figure S1B; Supplementary Table S1) (Schaniel et al., 2021). The comparisons between White and Asian showed 56 molecular signatures that significantly differed in combined male and female RNA-seq data (Figure 2A). Between the male and female hiPSCs from this repository, there are 21 DEGs (Figure 2B). Gene Ontology and KEGG pathway enrichment analysis revealed that these DEGs converge to a variety of molecular functions, cellular components, and biological processes (Supplementary Table S2; Supplementary Figure S2).

### Transcriptomics profiles in cardiomyocytes derived from hiPSCs of different races and sexes

Cardiovascular disease is the leading cause of death, and hiPSC-CM models have been used in disease modeling and therapeutic development. To understand the potential biological basis of cardiological differences among races (Mensah et al., 2005; Wadhera et al., 2021), we looked into the bulk RNA-seq transcriptomics profiles of cardiomyocytes derived from normal hiPSCs of different races (Schaniel et al., 2021). Intriguingly, there were 484 DEGs between female and male White hiPSC-CMs (Figure 2C). Gene Ontology and KEGG pathway enrichment analysis revealed that these DEGs of different hiPSC-CMs also converge to a variety of molecular functions, cellular components, and biological processes (Supplementary Table S3; Supplementary Figure S3).

Using the Maximum relevance minimum redundancy (mRMR) algorithm (see Methods), we sorted these DEGs based on their Importance Score (Figure 2D). We implemented several machine learning classifiers and tested the prediction accuracy of the ten mRMR genes with an accuracy >0.90, suggesting the novel biomarker potential of the hub genes related to cardiovascular disease and diabetes (Supplementary Figure S3B; Supplementary Table S4). The ten hub genes with the highest importance score of the mRMR algorithm were used for TF-miRNA-mRNA interaction network analysis. Notes, for the ten hub genes from the comparison between White male and female hiPSC-CMs, we found a sub-regulatory network that centered on miRs (miR-27, miR-26), and TFs (*FOXL1, NFIC, YY1, USF1, FOXC1, and GATA2*), and these important miRNAs, TFs interact with hub genes (*DDX3Y, EIF1AY, USP9Y, PRKY, TXLNGY, and UTY*) (Supplementary Figure S3C).

### Difference of single-cell transcriptomic profiles in hiPSC-CMs of different sexes

**FIGURE 3:**
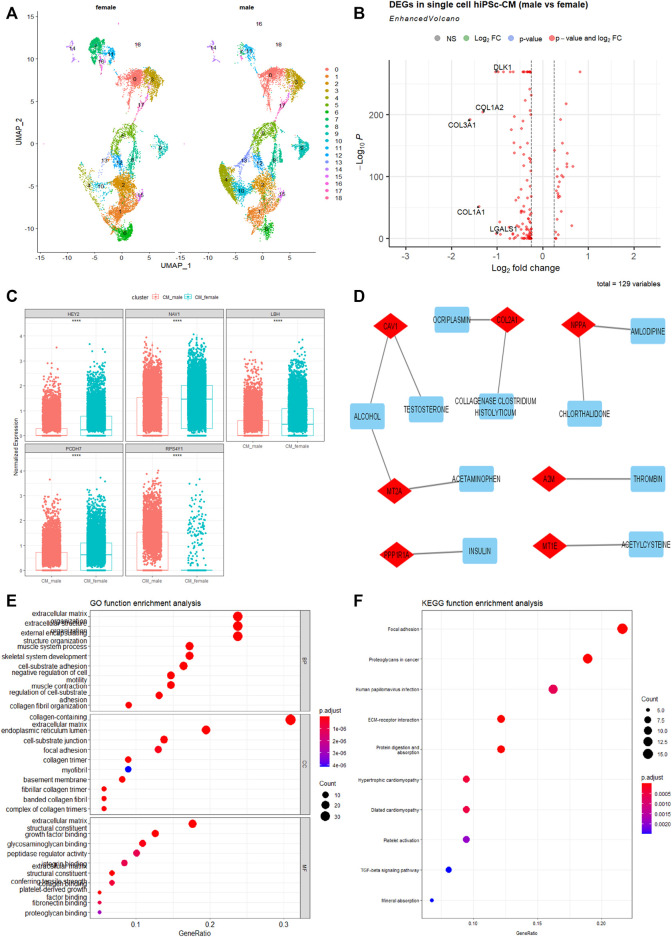
Single-cell transcriptomics differences between cardiomyocytes derived from hiPSC (hiPSC-CMs) of male and female White. **(A)** The UMAP projects 19 clusters of cardiomyocytes derived from 2 male and 2 female health hiPSC lines. Each cluster was labeled with an Arabic number. **(B)** Volcano map of DEGs between White male and White female hiPSC-CMs. **(C)** The top five hub genes in the best module significantly related to sex after WCGNA analysis using single-cell transcriptomics data. ****p < .0001. **(D)** Drug-genes interaction network derived from the sex-dependent DEGs in hiPSC-CMs. Drugs are denoted in blue, Drugs-genes interactions are in gray, and genes are in red. **(E)** Gene Ontology Enrichment analysis of the DEGs. **(F)** KEGG function enrichment analysis of the DEGs. The abscissa signifies the number of genes enriched in the function, and the ordinate is the name of the different types, which include molecular function, cellular components, and biological processes.

To further show sex as a crucial biological variable at the transcriptomics level, we analyzed the single-cell RNA-sequencing (scRNA-seq) data of cardiomyocytes derived from healthy White male and female hiPSCs of the same repository (Schaniel et al., 2021). As shown in Figure 3A, several clusters of hiPSC-CMs are different between males and females, with more cells in clusters 4, 10, and 13, and fewer cells in clusters 7 and 16 in male hiPSC-CMs compared to female ones. We further identified the overall DEGs between male and female hiPSC-CMs and found that genes associated with collagen formation and extracellular matrix (*COL1A1*, *COL1A2, and COL3A1*) are significantly lower in male hiPSC-CMs (Figure 3B).

To better understand gene molecular related to sex, this study constructed a gene co-expression network through the WGCNA R package (Langfelder and Horvath, 2008). We found twelve co-expression modules (Supplementary Figure S4) that contained from 37 to 989 genes screened. The most interesting module was the yellow module (r = −0.37, *p* < .0001). The genes in the yellow module overlap with DEGs, which may be potential hub genes (Figure 3C). GO and KEGG signaling pathway analyses show that these DEGs are enriched in focal adhesion, extracellular matrix, and are potentially important in cardiomyopathy processes (Figures 3E, F). A number of these genes can also interact with drugs, as revealed by the enriched Drug-Genes interaction network analysis (Figure 3D). These factors can be important targets in pathophysiological intervention and pharmaceutical development. Understanding the differences in gene expression between the sexes and races would be useful in developing more specific and effective therapeutics.

## Discussion

A large and diverse bank of iPSC is necessary for furthering biomedical research as these cell lines are used in disease modeling and pharmaceutical developments. The major hiPSC repositories state shows low diversity in ethnicity and disease models, and an imbalance in male and female hiPSC lines. There is an unequal distribution of iPSCs derived from different ethnicity. The White cohort makes up more than half of the total samples and is close to the recent 2021 US Census of 59.3%; and the next identified race, Black, comprises only a small fraction, 4.5% which is fewer than the recent 2021 Census estimate for the Black (13.6%). On the other hand, the “Other” ethnicity accounted for 30.9% of the total repository. As the repositories included in this analysis are mainly in the U.S. and Europe, it is expected that the hiPSCs identified with non-White ethnicity are fewer.

Nonetheless, besides strategically implementing a diverse ethnicity design in stem cell work, proper identification of race/ethnicity is essential to improve our knowledge of the biological basis, e.g., genetic ancestry (race and ethnicity) as an important factor in biomedical sciences and drug development (Schaniel et al., 2021). For disease types, the neural cohort made up the majority of the total hiPSCs samples. When both parameters are cross-analyzed, this study found that the White ethnicity comprises the majority of each disease/organ type except for blood and intestinal iPSC models. And neural disease models comprise a large portion of each ethnic group except for NHPI, who have more cardiovascular and diabetic diseases-related hiPSC samples. This data shows that there is a large discrepancy in ethnic groups and disease/organ types for the hiPSC resources as compared to the populations and the disease prevalence. This discrepancy may cause an ethnocentric and organ-centric research interest due to the different availability of hiPSCs. An active curation for “Other” ethnic groups [e.g., based on SNP Genotyping (Alexander et al., 2009)] and diseased organs will better reflect the state of hiPSC diversity.

The sex comparison shows a slight difference, with more male hiPSCs in the total repositories. In the sex analysis within each ethnic group, male samples comprised the majority of each group except for the NHPI group, which has a 50–50 ratio. And the analysis of the ethnic composition of each sex shows that the White ethnic group makes up the majority of each sex. Although this study found a slight difference in the total samples, when we look at each ethnic group separately, the difference in sexes is more pronounced. This is obvious in the American Indian and Alaska Native group, which is likely due to the low number of hiPSCs. During curation, there should be an effort to gather samples from both sexes, especially in the underrepresented ethnic groups.

From our molecular analysis of races, there are more hiPSCs of the White than of other races. The comparison between the races shows that non-differentiated hiPSCs are relatively comparable to each other as evident assessed by fewer DEGs between their transcriptomes (Figure 2A). Between the two sexes, there were multiple enriched pathways (Figures 2B, C, and Figure 3; Supplementary Figures S2B, S3A). The results showing fewer DEGs in hiPSCs than hiPSC-CMs between the two sexes, confirming that differentiated cells such as cardiomyocytes show significant sex-dependent molecular features that can contribute to pathophysiological differences between male and female hearts. Nonetheless, male and female pluripotent stem cells differ in autosomal gene expression as evident by transcriptomics analysis, albeit their comparable ground state of stemness (Ronen and Benvenisty, 2014). Cellular sex difference is a key factor in dimorphic pathophysiology, which is caused by both sex hormones and chromosomal genotype (Walker et al., 2021), as well as sex-dependent different regulations in genomic imprinting (Arez et al., 2022). Some genes that were differentially expressed were sex-specific; for example, Zinc Finger Y-linked (*ZFY*) and Taxilin Gamma (*TXLNGY*) (Figure 2). These genes are associated with gene regulation, acting as transcription factors and transcription inhibitors. Their function is not well defined in gene regulation, which makes it potential targets for future studies. We also found the Wnt pathway being more enriched in the male group (Supplementary Figure S2B). The Wnt pathway is widely associated with cell fate during embryonic development which could explain the differing transcriptomes. In addition, it was found that sex plays a large biological role in the development of central nervous system disorders due to the differences in gene expression (Kiris, 2022). This difference in transcriptome is also found in myeloid cells when iPSC were used as a model for Alzheimer’s (Coales et al., 2022). As stem cells are increasingly being used in modeling human cell development and disease processes as well as in clinical interventions (Tang et al., 2022) further investigation of the physio-pathological significance will help guide biomedical research and therapeutic development that are inclusive of ethnicity and sex.

There are some limitations in this study such as the large population of other/unidentified samples. We also recognize that the repositories included in this analysis were only at our extent of getting the American and European ones. In addition to those included in the full cross-matrix analysis (Figure 1), we received hiPSC repository data from New York Stem Cell Foundation for limited analysis, which shows similar level of a lack of diversity (Supplementary Figure S5). Future attempt should include the analysis of hiPSC repositories of other regions. In addition, the analysis is based on the assumption that hiPSCs have comparable stemness, and cardiac differentiation from hiPSCs of each line is equally efficient. While the sample sizes in transcriptomics analysis for each race and sex may be small, the data in hiPSC-CMs (bulk RNA-seq, and scRNA-seq) and hiPSCs suggested the differences are very plausible. Non-etheless, we could not exclude the possibility that any residual epigenetic backgrounds from the source tissues (cells) for hiPSC lines affect transcriptomes of their derived cardiomyocytes even if they present comparable functions (Xu et al., 2012). Lastly, while our transcriptomics analysis showed that DEGs between hiPSCs and their derived CMs can be protein-coding or non-coding (e.g. long non-coding RNA; Figure 2C; Supplementary Figure S3C), analysis at protein level difference will reveal better patterns that closely reflect functional differences between stem cells and differentiated cells of different races and sexes.

In conclusion, the data shows current iPSC banks in North America and Europe lack diversity with respect to ethnicity, sex, and disease/organ modeling. Active curation of hiPSC lines and obtaining more samples from different organ types and unrepresentative ethnicities will increase the diversity of the repository. This shall close the gap of diversity in stem cell research and enhance the precision designs in disease modeling and pharmaceutical developments.

## Data Availability

The original contributions presented in the study are included in the article/Supplementary Material, further inquiries can be directed to the corresponding author.
