# Supplementary material for: Harshening stem cell research and precision medicine: The states of human pluripotent cells stem cell repository diversity, and racial and sex differences in transcriptomes
Source: Front Cell Dev Biol. 2023 Jan 4;10:1071243. doi: 10.3389/fcell.2022.1071243 (PMC9848738; doi:10.3389/fcell.2022.1071243)
Supplement: Supplementary file 1 [file DataSheet1.pdf]

SUPPLEMENTAL INFORMATION

for

Harshening Stem Cell Research and Precision Medicine: the States of Human Pluripotent Stem Cell Repository Diversity and Racial and Sex Differences in Transcriptomes

Thong Ba Nguyen[1,2,*], Quan Lac[1,2,*], Lovina Abdi[1,2], Dipanjan Banerjee,[2,3] Youping Deng[4, 5], Yiqiang Zhang[1,2,6¶]

[1]Department of Anatomy, Biochemistry and Physiology, [2]Center for Cardiovascular Research, [3]Department of Medicine, [4]Department of Quantitative Health Sciences, [5]Genomics and Bioinformatics Shared Resource, [6]Diabetes Research Center, John A. Burns School of Medicine, University of Hawaii at Manoa, Honolulu, HI, USA

* Equal contributions.

¶ Correspondence should be addressed to: Dr. Yiqiang Zhang, Department of Anatomy, Biochemistry and Physiology, John A. Burns School of Medicine, University of Hawaii, Honolulu, HI, 96813, USA; e-mail: Yiqiang.Zhang@hawaii.edu.

SUPPLEMENTAL INFORMATION:

I. **Supplemental Figures and Figure Legends (5)**

II. **Supplemental Tables (5)**

I. Supplement Figures and Legends

Figure S1.

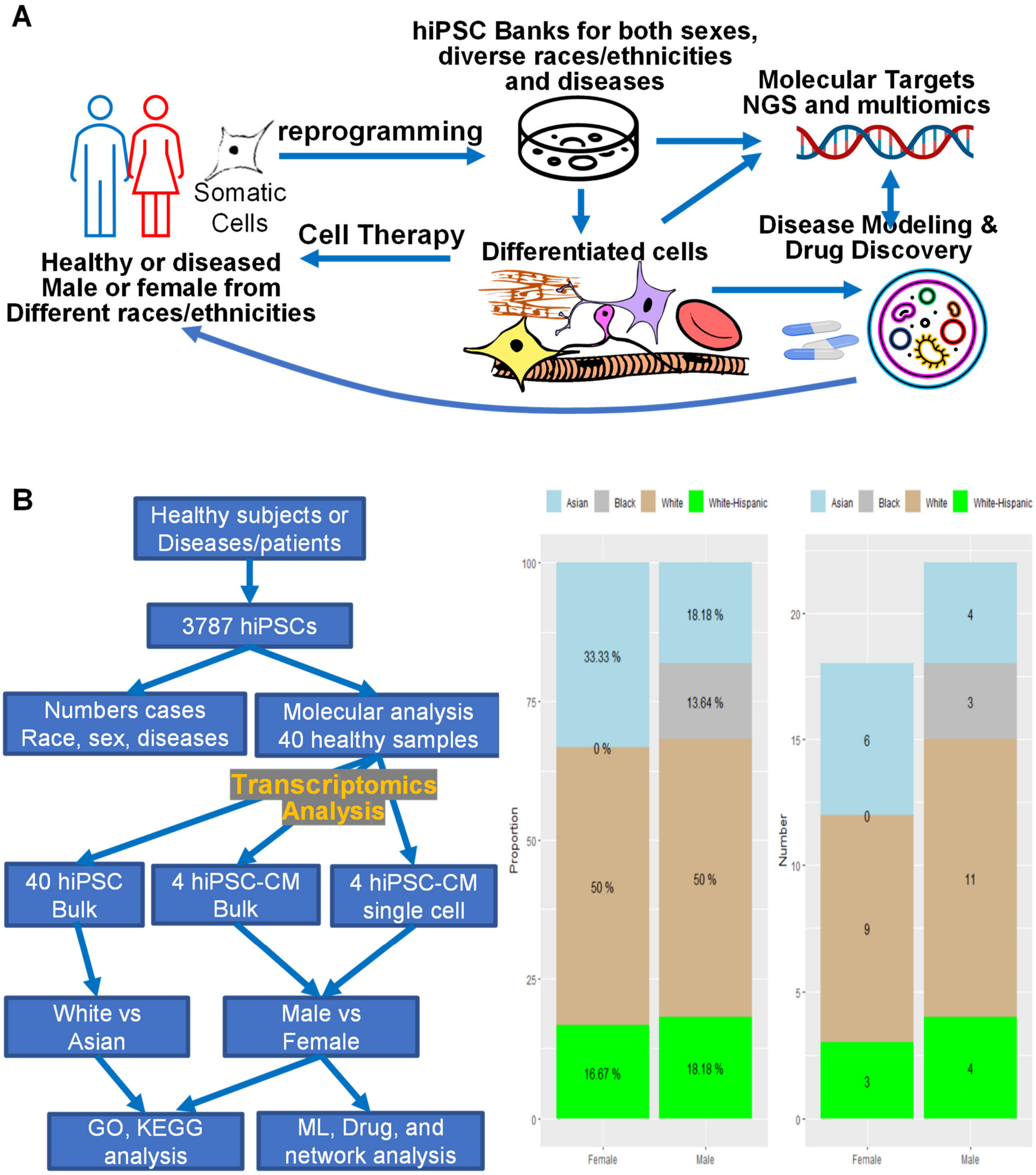

Figure S1. (**A**) General workflow of hiPSC R&D programs involving different races and sexes from whom somatic cells are obtained for reprogramming into induced pluripotent stem cells (hiPSCs). hiPSCs can be differentiated into various cells for cell therapeutics, diseases modeling and drug discovery. Both hiPSCs and differentiated cells and model systems can be analyzed with comprehensive molecular assays such as next generation sequencing (NGS). (**B**) Transcriptomics analysis data sources and workflow (left) and the composition of the relatively balanced diversity panel of healthy hiPSC from Mont Sinai Med Center database (right).

Figure S2.

A

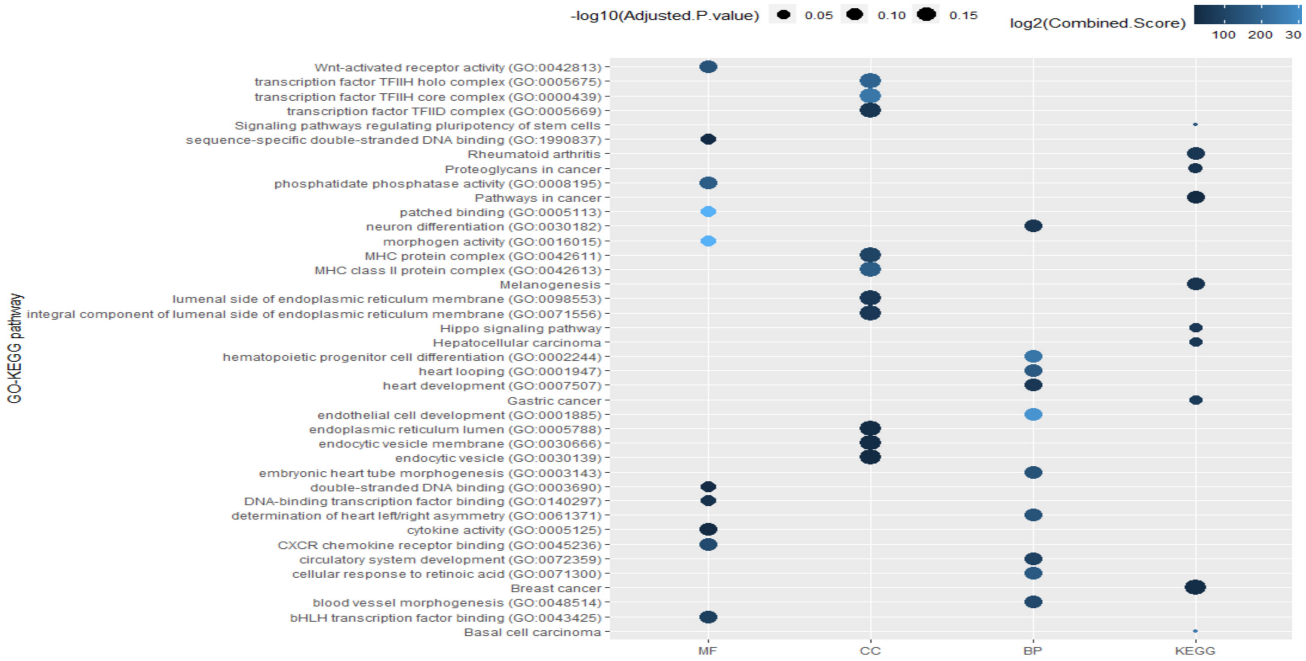

B

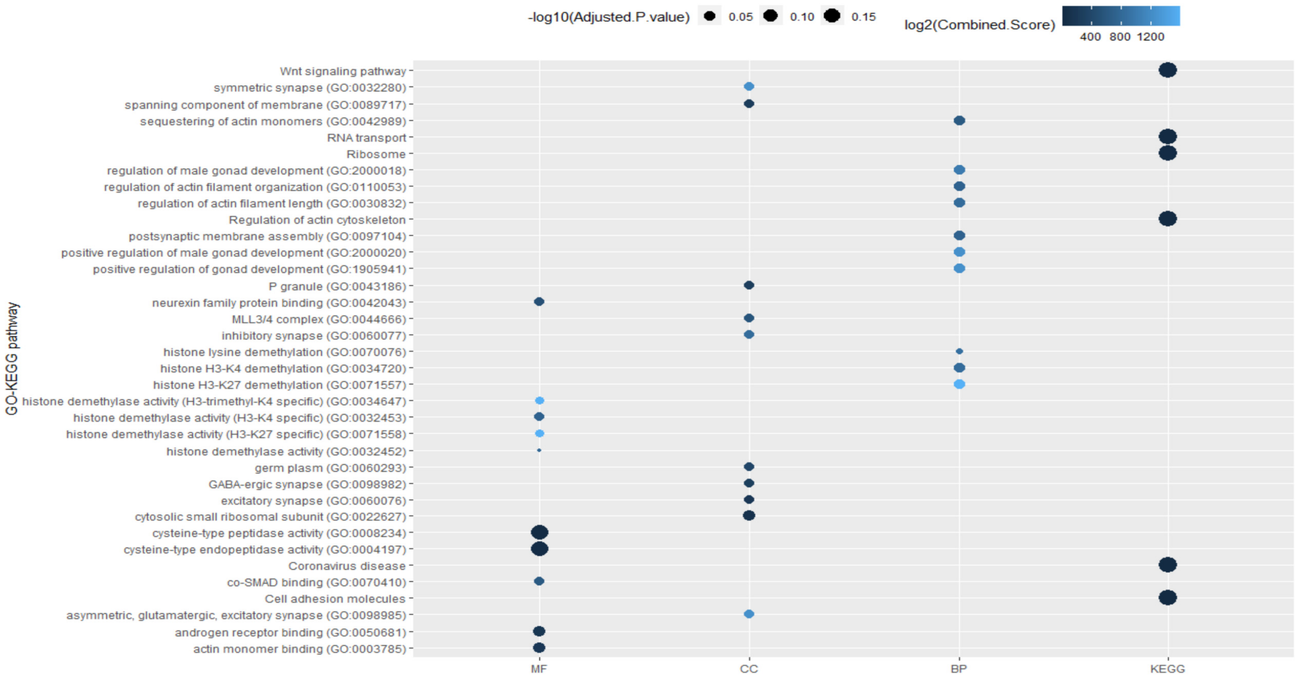

Figure S2. GO and KEGG functional analysis of DEGs from hiPSC data (related to Figure 2A, 2B). **(A)** GO and KEGG of DEGs between White and Asian hiPSCs (combined male and female). (**B**) GO and KEGG of DEGs between males and females from combined White, Asian, and Hispanic datasets. The intensity of the color in the dotplot indicates the enrichment significance by the log2 combined score. Circle sizes correspond to the -log10 adjusted p-value (padj). Gene set names are ordered according to the GO molecular function (MF), cellular components (CC), biological processes (BP), and Kyoto Encyclopedia of Genes and Genomes (KEGG).

Figure S3.

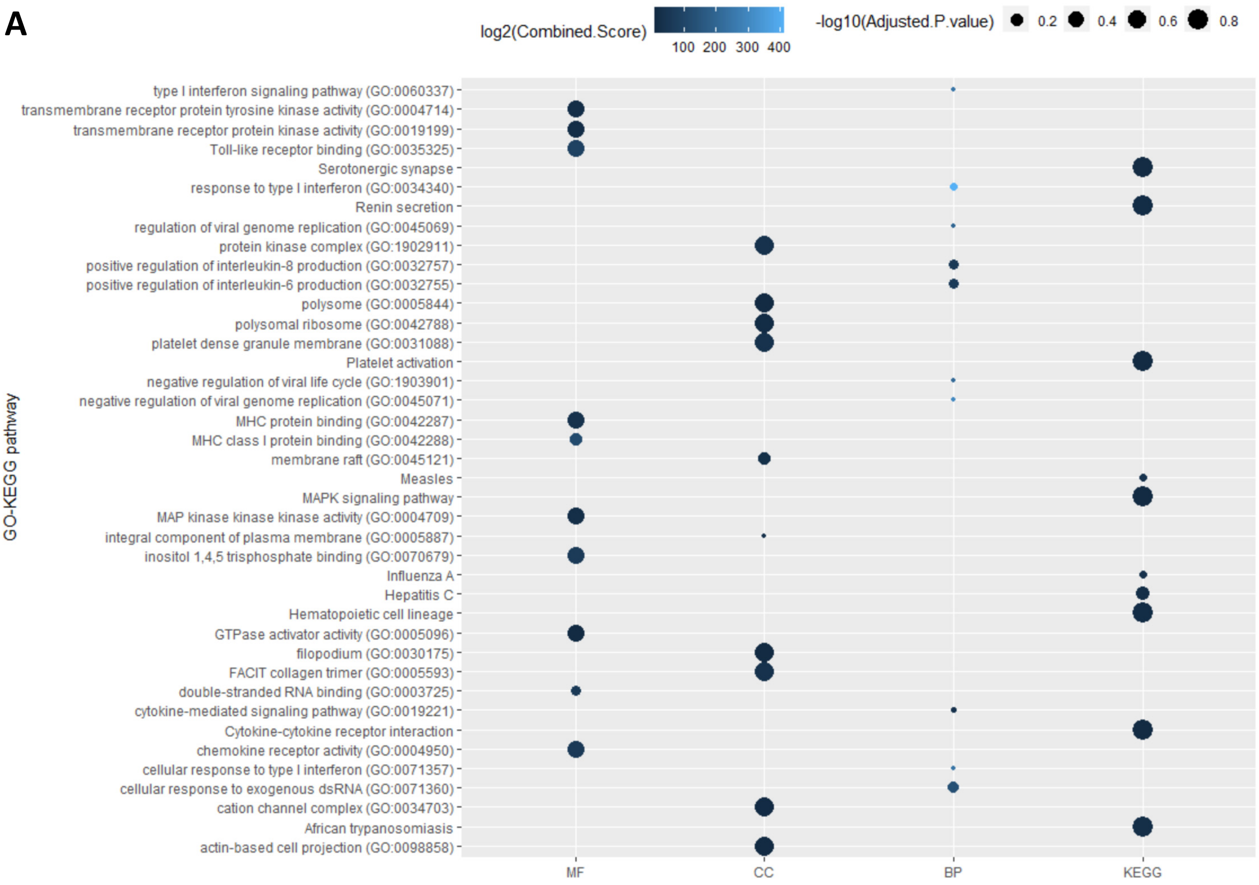

Figure S3. GO and KEGG functional analysis of DEGs from hiPSC-CM data (related to Figure 2B, 2C). **(A)** GO and KEGG of DEGs between White males and White females. The intensity of the color in the dotplot indicates the enrichment significance by the log2 combined score. Circle sizes correspond to the -log10 adjusted p-value (padj). Gene set names are ordered according to the GO molecular function (MF), cellular components (CC), biological processes (BP), and Kyoto Encyclopedia of Genes and Genomes (KEGG).

Figure S3.

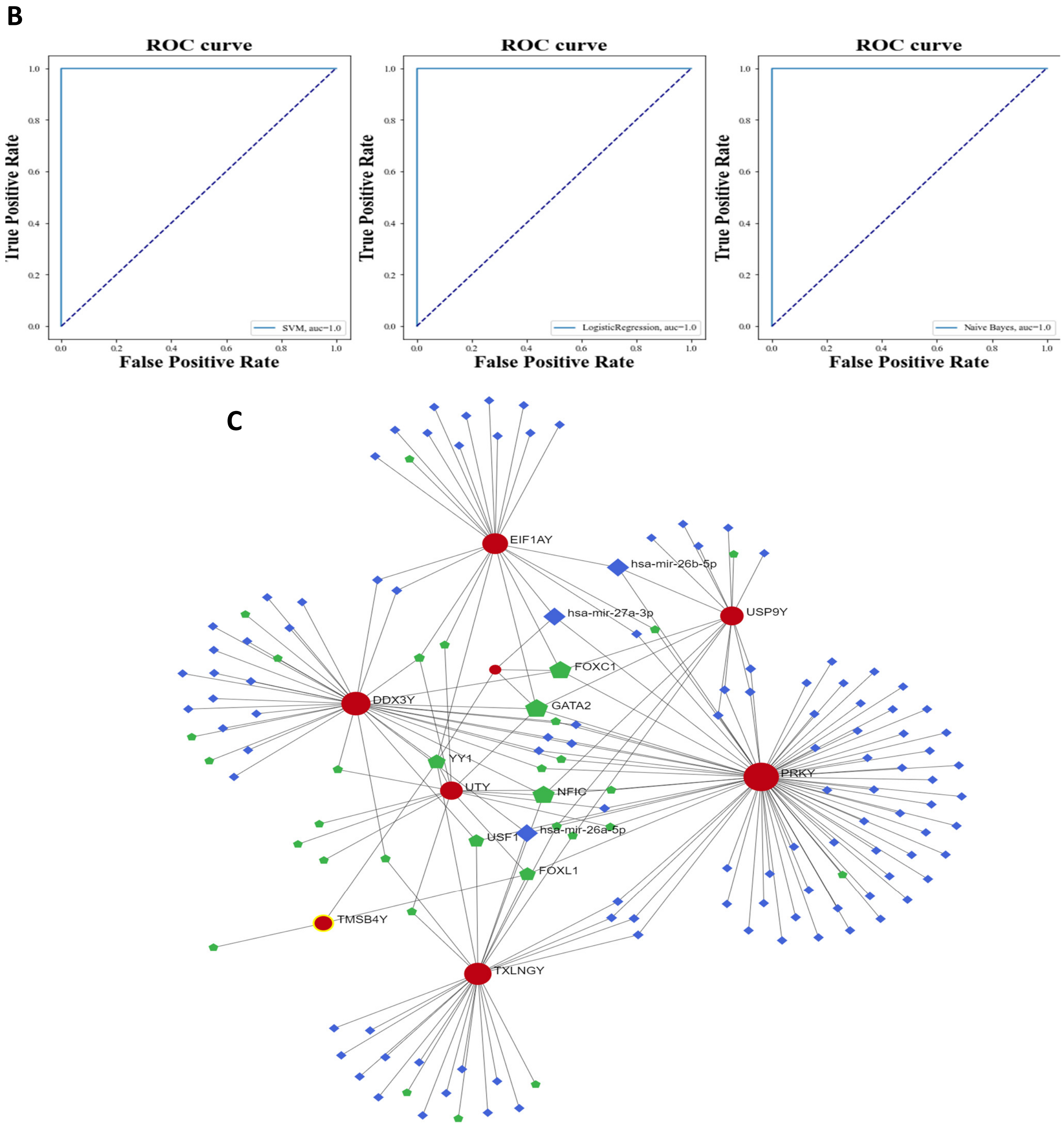

Figure S3. GO and KEGG functional analysis of DEGs from hiPSC-CM data (related to Figure 2). Algorithm classification analysis and regulatory network of TF-miRNA-mRNA of ten hub genes between White males and White females received from hiPSC-CM data after mRMR selection. **(B)** Receiver operating characteristic (ROC) curve of the three classifier performances based on AUC. **(C)** Regulatory network of hub genes.

Figure S4

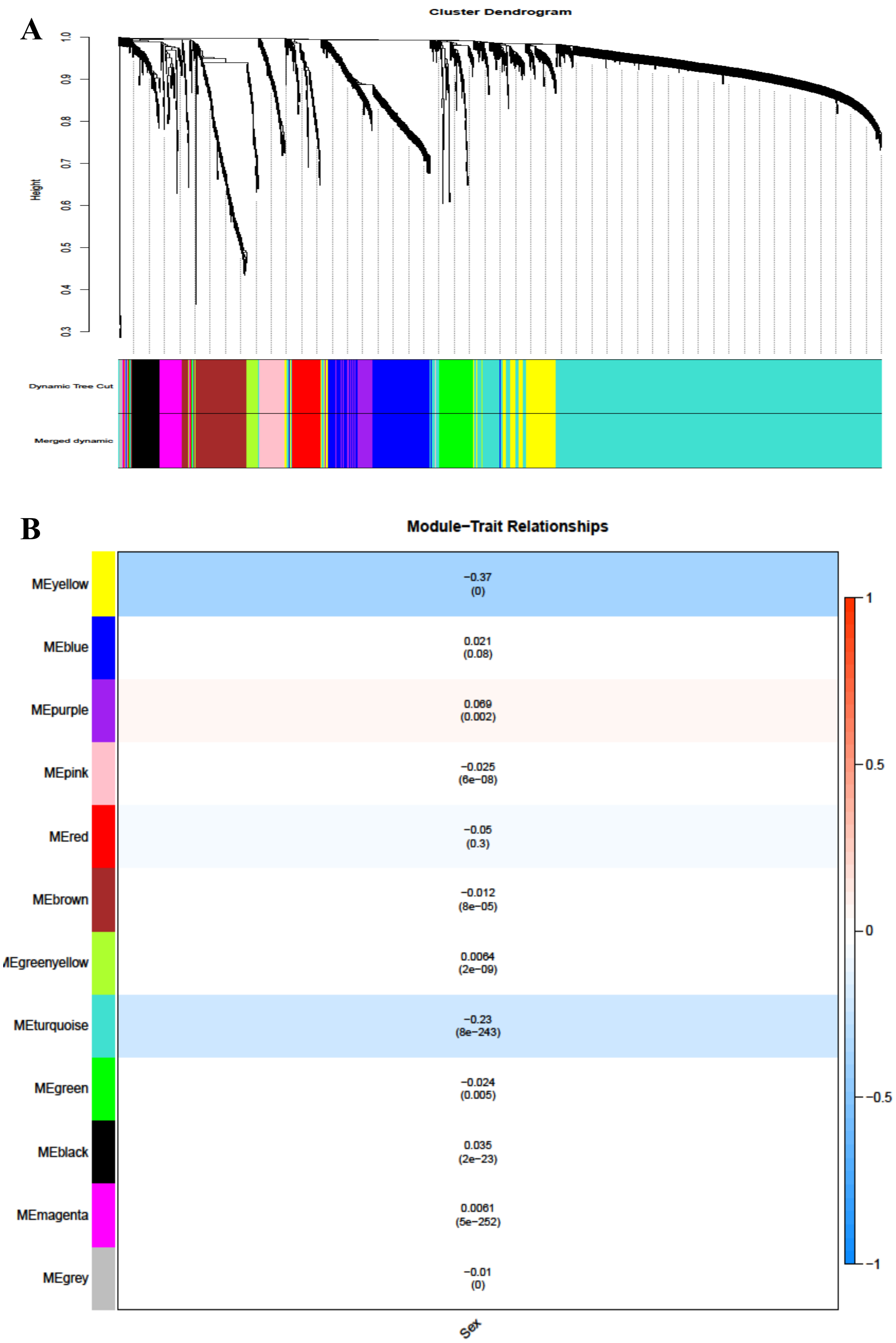

Figure S4: (Related to Figure 3). Gene co-expression network analysis. **(A)** A gene clustering tree (cluster dendrogram) is obtained by hierarchical clustering of adjacency-based dissimilarity, each color represents a module, and each vertical line represents a gene. **(B)** Module-trait relationships: representing the correlation and p-value (in parentheses) between each module eigengene (ME) and sex phenotype.

Figure S5

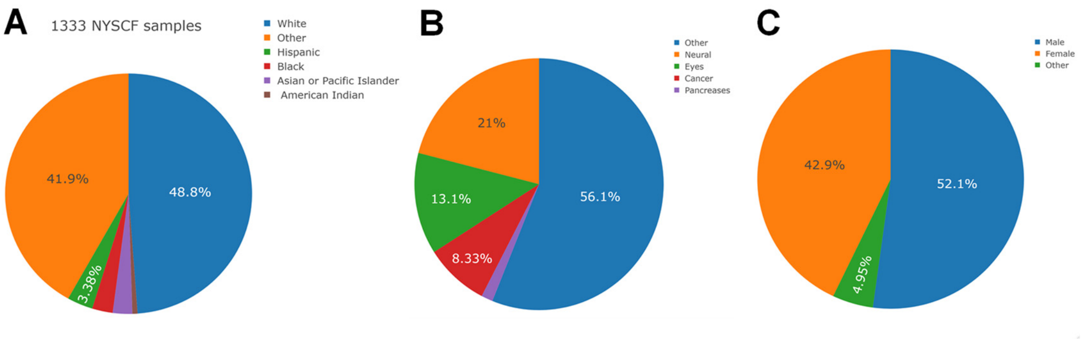

Figure S5. Compositions of hiPSC lines in NYCSF database. **(A)** Distribution of races and ethnicity of hiPSC lines. **(B)** distribution of diseases of hiPSC lines. **(C)** Distribution of sex of hiPSC lines.

II. Supplemental Tables

Table S1. Sample composition in bulk- and single-cell RNA-seq analysis (Schaniel et al., 2021).

Race	hiPSC (bulk RNA-seq)		hiPSC-CMs (bulk RNA-seq)		hiPSC-CMs scRNA-seq	
	Male	Female	Male	Female	Male	Female
White	11	9	2	2	2	2
Black	3					
Asian	4	6		1		
Hispanic	4	3	1			

Table S2. Summary of the identified 10 genes in each dataset in hiPSC.

**White vs Asian(n=20;10)**			**Male vs Female (n=19;18)**		
Gene name	Log2(FC)	Adj. P-value	Gene name	Log2(FC)	Adj. P-value
TMEM132D	-3.614	0.035	RPS4Y1	8.51	<0.001
LOC284581	3.603	0.031	USP9Y	8.136	<0.001
HLA-DPB1	-3.159	0.035	EIF1AY	7.862	<0.001
GTF2H4	-2.963	0.041	TXLNGY	7.779	<0.001
COL22A1	-2.722	0.046	TTTY15	7.349	<0.001
GLT1D1	-2.669	0.044	UTY	7.244	<0.001
CHMP1B2P	-2.455	0.044	DDX3Y	6.938	<0.001
HCG18	-2.287	0.044	TMSB4Y	6.729	<0.001
LOC107987388	-2.267	0.009	NLGN4Y	6.503	<0.001
ADAMTS7P1	-1.979	0.044	XIST	-6.427	<0.001

Table S3. Summary of the identified 10 genes in each dataset in hiPSC-CMs (related to Figure 3).

**Between white male vs female (n=2;2)**		
Gene name	Log2(FC)	Adj. P-value
DDX3Y	10.627	<0.001
USP9Y	10.612	<0.001
EIF1AY	10.044	<0.001
UTY	9.634	<0.001
PRKY	9.261	<0.001
TXLNGY	8.994	<0.001
ZFY	8.53	<0.001
TMSB4Y	6.77	<0.001
LOC101927999	6.329	<0.001
LOC105377223	5.934	<0.001

Table S4. Overview of top 3 hub proteins related to DM and CVD in each hiPSC-CM dataset (related to Figure 2, and S3)

**Comparison**	**Symbol**	**Description**	**Aspect (relate to diabetes or heart failure)**	**References**
**Between White male vs female (n=2;2)**	**DDX3Y**	DEAD-box helicase 3 Y-linked	Regulates adult somatic cell (Cardiac) functions with Uty/Ddx3y axis	(Deschepper, 2020)
	**USP9Y**	Ubiquitin Specific Peptidase 9 Y-Linked	Ubiquitin-Proteasome Dependent Proteolysis: unregulated in ischemic cardiomyopathy	(Li et al., 2018)
	**EIF1AY**	Eukaryotic Translation Initiation Factor 1A, Y chromosome	Over expression during onset of heart failure	(Heidecker et al., 2010)

Table S5. Top regulatory signatures predicted from DEG-TF-miRNA interaction networks (related to Figure 2C, 2D).

Comparison	Symbol	Description	Aspect (relate to diabetes or heart failure)	References
**Between white male vs female (n=2;2)**	**miR-26**	MicroRNA 26	Central role in cardiovascular disease by controlling critical signaling pathways such as BMP/SMAD1 signaling and target relevant to left ventricle function after MI	(Icli et al., 2014)
	**miR-27**	MicroRNA 27	Promotes miR-27 promotes cardiac fibrosis by impacting the Snail pathways	(Fu et al., 2019)
	**FOXL1**	Forkhead Box L1	Elevated expression is associated with good outcomes in pancreatic ductal adenocarcinoma, but no know association with cardiac diseases.	(Haidar et al., 2020)
	**NFIC**	Nuclear Factor I C	Participates on ischemic cardiomyopathy process by regulating TSPAN1 and HOPX	(Ye et al., 2022)
	**YY1**	Yin Yang 1 Transcription Factor	Interacts with the alpha Myosin Heavy Chain promoter to repress the activity of promoter	(Sucharov et al., 2003)
	**USF1**	Upstream Transcription Factor 1	USF1 is a critical transcription factor regulating diabetic kidney disease and plays a critical role in albuminuria, mesangial matrix accumulation, and TGF-β1 and renin stimulation in diabetic kidney disease.	(Sanchez et al., 2011)
	**FOXC1**	Forkhead Box C1	Directly targets Myh7 expressed during cardiac development and functional properties in ESC-derived cardiomyocytes	(Lambers et al., 2016)
	**GATA2**	GATA Binding protein 2	Transcription factor through convergent analysis of linkage and expression data to define mechanisms that lead to CAD	(Connelly et al., 2006)

References

Connelly, J.J., Wang, T., Cox, J.E., Haynes, C., Wang, L., Shah, S.H., et al. (2006). GATA2 is associated with familial early-onset coronary artery disease. *PLoS Genet 2*, e139. doi: 10.1371/journal.pgen.0020139

Deschepper, C.F. (2020). Regulatory effects of the Uty/Ddx3y locus on neighboring chromosome Y genes and autosomal mRNA transcripts in adult mouse non-reproductive cells. *Sci Rep 10*, 14900. doi: 10.1038/s41598-020-71447-3

Fu, Q., Lu, Z., Fu, X., Ma, S., and Lu, X. (2019). MicroRNA 27b promotes cardiac fibrosis by targeting the FBW7/Snail pathway. *Aging (Albany NY) 11*, 11865-11879. doi: 10.18632/aging.102465

Haidar, M.N., Islam, M.B., Chowdhury, U.N., Rahman, M.R., Huq, F., Quinn, J.M.W., et al. (2020). Network-based computational approach to identify genetic links between cardiomyopathy and its risk factors. *IET Syst Biol 14*, 75-84. doi: 10.1049/iet-syb.2019.0074

Heidecker, B., Lamirault, G., Kasper, E.K., Wittstein, I.S., Champion, H.C., Breton, E., et al. (2010). The gene expression profile of patients with new-onset heart failure reveals important gender-specific differences. *Eur Heart J 31*, 1188-1196. doi: 10.1093/eurheartj/ehp549

Icli, B., Dorbala, P., and Feinberg, M.W. (2014). An emerging role for the miR-26 family in cardiovascular disease. *Trends Cardiovasc Med 24*, 241-248. doi: 10.1016/j.tcm.2014.06.003

Lambers, E., Arnone, B., Fatima, A., Qin, G., Wasserstrom, J.A., and Kume, T. (2016). Foxc1 Regulates Early Cardiomyogenesis and Functional Properties of Embryonic Stem Cell Derived Cardiomyocytes. *Stem Cells 34*, 1487-1500. doi: 10.1002/stem.2301

Li, Y., Jiang, Q., Ding, Z., Liu, G., Yu, P., Jiang, G., et al. (2018). Identification of a Common Different Gene Expression Signature in Ischemic Cardiomyopathy. *Genes (Basel) 9*. doi: 10.3390/genes9010056

Sanchez, A.P., Zhao, J., You, Y., Declèves, A.E., Diamond-Stanic, M., and Sharma, K. (2011). Role of the USF1 transcription factor in diabetic kidney disease. *Am J Physiol Renal Physiol 301*, F271-279. doi: 10.1152/ajprenal.00221.2011

Schaniel, C., Dhanan, P., Hu, B., Xiong, Y., Raghunandan, T., Gonzalez, D.M., et al. (2021). A library of induced pluripotent stem cells from clinically well-characterized, diverse healthy human individuals. *Stem Cell Reports 16*, 3036-3049. doi: 10.1016/j.stemcr.2021.10.005

Sucharov, C.C., Mariner, P., Long, C., Bristow, M., and Leinwand, L. (2003). Yin Yang 1 is increased in human heart failure and represses the activity of the human alpha-myosin heavy chain promoter. *J Biol Chem 278*, 31233-31239. doi: 10.1074/jbc.M301917200

Ye, Y., Jin, Q., Gong, Q., Li, A., Sun, M., Jiang, S., et al. (2022). Bioinformatics and Experimental Analyses Reveal NFIC as an Upstream Transcriptional Regulator for Ischemic Cardiomyopathy. *Genes (Basel) 13*. doi: 10.3390/genes13061051
